# Sugar, Spice, and Bleeding

**DOI:** 10.7759/cureus.4437

**Published:** 2019-04-11

**Authors:** Zainab Shahid, Ricci Kalayanamitra, Maria Hanafi, Khurram Anwar, Rohit Jain

**Affiliations:** 1 Internal Medicine, Lake Erie College of Osteopathic Medicine, Erie, USA; 2 Emergency Medicine, Penn State Health Milton S. Hershey Medical Center, Hershey, USA; 3 Internal Medicine, Atlantic Health System, Morristown Medical Center, Morristown, USA; 4 Internal Medicine, Penn State Health Milton S. Hershey Medical Center, Hershey, USA

**Keywords:** long-acting anticoagulant rodenticide, superwarfarin, synthetic cannabinoid, bleeding, brodifacoum, coagulopathy

## Abstract

Brodifacoum (BDF), otherwise known as superwarfarin, is a long-acting anticoagulant rodenticide (LAAR) which acts as a vitamin K antagonist. Much like warfarin, BDF’s mechanism of action is to irreversibly inhibit the enzyme vitamin K epoxide reductase, thus reducing the recycling of vitamin K and, therefore, reducing the activation of clotting factors two, seven, nine, and 10. Although BDF is usually found in rodenticides, it has been recently used as an additive in synthetic cannabinoids. We present a case of a young male with a history of synthetic cannabinoid use who presented with hematuria and epistaxis and was ultimately found to have BDF poisoning.

## Introduction

Brodifacoum was initially introduced in rodenticides in 1975 in order to manage the growing problem of warfarin-resistant rodents [1]. It exists as cis- and trans-enantiomers of 4-hydroxycoumarin. BDF-cis is significantly more toxic than BDF-trans, and racemic BDF has intermediate potency [2]. In the past, patients with BDF poisoning presented due to accidental exposure to rodenticides, psychosis leading to rodenticide ingestion, and deliberate rodenticide ingestion during suicide attempts [3-5]. Recently, patients have been presenting with BDF poisoning due to synthetic cannabinoid use.

Although synthetic cannabinoids were originally developed to study the function of cannabinoid receptors, they have been used recreationally in the USA since 2008 and are sold under names such as “K2” and “Spice.” Many patients have been using synthetic cannabinoids because these particular drugs are not detectable on standard drug screens, including tetrahydrocannabinol assays, but induce euphoria that is reportedly similar to that of marijuana. There are a few commercially available immunoassay kits that can detect synthetic cannabinoids, such as the DrugCheck K2/Spice test, but they do not detect all synthetic cannabinoids [6].

The most common side effect of BDF poisoning due to synthetic cannabinoid use is bleeding and the most frequently reported sites of bleeding are mucocutaneous, with hematuria being the most reported. Deaths caused by BDF-related coagulopathy are most commonly due to intracranial hemorrhage [7]. Due to these dangerous side effects, the US federal government passed the Synthetic Drug Abuse Prevention Act of 2012, which permanently designated synthetic cannabinoids as Schedule I controlled substances [8]. The Centers for Disease Control and Prevention (CDC) recently issued a warning for synthetic cannabinoids contaminated with BDF which have been on the drug market since 2018. The first case of hypocoagulability associated with synthetic cannabinoid use was identified in March of 2018 in Illinois, and since then at least 150 patients have presented with bleeding due to confirmed BDF exposure across 11 states according to the CDC. As of December 2018, there were at least eight reported fatalities [9].

## Case presentation

A 22-year-old male with no significant past medical history and a five-year history of synthetic cannabinoid use presented to the emergency department with complaints of hematuria and epistaxis. The patient had stable vital signs, and his physical examination revealed a right conjunctival hemorrhage, active epistaxis of the left nostril, and blood-tinged urine. Laboratory workup revealed a prothrombin time (PT) greater than 106 s and a partial thromboplastin time (PTT) of 79.5 s. His international normalized ratio (INR) was not determinable.

The patient admitted to adding rodenticides to his synthetic cannabinoids in order to increase their euphoric effect. He was subsequently admitted to the hospital for monitoring and management of blood loss in the setting of an acute chemical-induced coagulopathy. He was given an initial dose of intravenous vitamin K1 50 mg and the poison control center was notified. The patient then received two doses of oral vitamin K1 50 mg over the next two days until his PT normalized.

## Discussion

Brodifacoum poisoning due to synthetic cannabinoid use has become an increasingly significant concern in the USA. This case highlights the major nonpsychotropic effect of BDF-contaminated synthetic cannabinoids, which is bleeding. In BDF poisoning, bleeding is prolonged and can be fatal at high doses due to BDF’s high lipophilicity and long half-life. In one study, BDF’s elimination half-life was estimated to be over 90 days in plasma and approximately 10 months in the liver [10]. The prolonged half-life of BDF necessitates extended courses of treatment with high-dose vitamin K1 supplementation. One study of over 315,000 patients with exposure to LAARs between 1987 and 2012 revealed that chronic maintenance therapy with 100 mg of daily oral vitamin K1 was the most frequently used dose required to suppress coagulopathy and that the average treatment time was 168 days [7]. This is currently the only treatment method for BDF poisoning and it is very expensive, which can result in poor adherence by patients. Therefore, patients may have medication noncompliance and have recurrent bleeding events requiring further hospitalizations and prolonged treatment.

Although the mainstay of treatment for BDF poisoning is high-dose vitamin K1 supplementation, this method only restores coagulation and has no effect on BDF metabolism or clearance. Therefore, BDF remains in the patient’s bodies for the entire duration of its course and leaves them at risk for bleeding recurrence. In addition, BDF crosses the placenta and thus poses an additional risk for pregnant women who have been exposed [11]. This poses a significant public health concern as well, as the Food and Drug Administration (FDA) has released a statement that there may be possible contamination of the US blood supply as there have been several reports of blood donors who have used these contaminated drugs [12]. With the increasing incidence of cases of BDF poisoning due to synthetic cannabinoid use, more effective medications must be developed which directly affect the clearance or metabolism of BDF. Patients should be cautioned against using synthetic cannabinoids because it is not possible to detect which batches have been contaminated. In addition, patients who are on long-term treatment with vitamin K1 should be educated on the importance of treatment adherence and be advised against blood donation, and women of child-bearing age should be counseled on contraception use during their period of treatment.

## Conclusions

Given the rise in BDF-contaminated synthetic cannabinoids and the lack of an effective and affordable treatment for BDF poisoning, we recommend that patients be advised against using synthetic cannabinoids due to the potentially fatal adverse effect of bleeding. In addition, patients who have been diagnosed with BDF poisoning should be educated on the importance of treatment adherence and the usage of contraception and be counseled against blood donation.
